# Pre-hospital emergency cricothyrotomy in dogs part 2: Airway sealing and ventilation using cricothyrotomy tubes

**DOI:** 10.3389/fvets.2023.1129462

**Published:** 2023-02-17

**Authors:** Sureiyan Hardjo, Mark Haworth, Catriona Croton, Sarah Purcell, Wendy Goodwin

**Affiliations:** Faculty of Science, School of Veterinary Science, The University of Queensland, Gatton, QLD, Australia

**Keywords:** cricothyrotomy, H&H, Portex, military working dog, operational K9, CICO, airway—obstruction, tracheostomy

## Abstract

Cricothyrotomy (CTT) has been recommended for use in the pre-hospital setting for military working dogs and Operational K9s during airway emergencies. Although the CTT can establish a patent airway for spontaneous ventilation, the ability to seal the airway and provide positive pressure ventilation (PPV) using tubes designed for humans has not been determined. Using various CTT tubes placed in cadaver dog airways, this study aimed to determine: (1) Whether the tube cuff could create a functional airway seal with safe intra-cuff pressures; (2) The magnitude of delivered tidal volume (TV) loss during a standard breath to assess the possibility of delivering an adequate tidal volume with a bag-valve device (BVM); (3) The best performing tubes for either test; (4) The reasons behind the findings using observations from upper airway endoscopy, dissection, and measurements. Cadaver dogs of similar weights to MWD and Operational K9 breeds had various CTT tubes placed including three from commercial kits, a standard endotracheal tube, and a tracheostomy tube. The minimum occlusive volume technique was used to inflate the tube cuff and a pressure ≤ 48 cm H_2_O with an adequate seal was considered successful. Individual TVs were calculated for each dog and added to the volume lost during delivery of a standard breath from an ICU ventilator. Endoscopy and airway dissection were performed to assess the relationship between tubes cuffs and the airway. The tubes from the CTT kits performed poorly with regards to producing an airway seal with the H&H tube failing to seal the airway all tests. Tracheal dimensions were significantly associated with successful airway sealing (*P* = 0.0004). Tidal volume loss could be compensated using a BVM in 34/35 tests with the H&H tube in cadaver 8 the only to fail. Tracheal airway sealing is influenced by airway anatomy when tube cuffs are inflated to a target pressure and larger tubes do not always provide a better seal. The CTT tubes tested have the potential to facilitate ventilation with a BVM under the conditions set in this study. The 8.0 mm endotracheal tube performed the best and the H&H the worst in both tests.

## Introduction

The surgical tube cricothyrotomy (CTT) has been recommended for emergency front-of-neck airway access (eFONA) in military working dogs (MWD) and Operational K9s during a cannot intubate, cannot oxygenate (CICO) emergency (1, 2). Combat medics and human health care providers (HCP) are expected to perform first aid and emergency medical intervention for injured MWD (3). Cricothyrotomy kits are commonly carried by combat medics which typically contain 6.0 mm internal diameter (I.D.) tubes (4). However, size 11–14 mm I.D. endotracheal tubes are recommended for dogs of ~30 kg body weight (5). Hence, the efficacy of providing a functional airway seal between the tube cuff and tracheal mucosa in MWD breeds has been questioned (1). Ineffective airway sealing may result in two main complications:

1. Potential for aspiration of foreign material or refluxed ingesta.
2. Reduced efficacy of positive pressure ventilation (PPV).

Endotracheal tube intra-cuff (IC) pressures >48 cm H_2_O have the potential to reduce mucosal blood flow and mucosal loss can occur at pressures exceeding 67.5 cm H_2_O for 15 min (6, 7). Consequently, recommended IC pressures range between 20 and 30 cm H_2_O to prevent tracheal irritation and mucosal damage (6, 8). Appropriate endotracheal tube cuff inflation can be challenging in the prehospital setting due to lack of specialized equipment (8). Hence the first aim of this study to ascertain if CTT tube cuffs can produce a seal with an IC pressure of 48 cm H_2_O or less using the minimal occlusive volume (MOV) technique.

Cricothyrotomy is capable of facilitating oxygenation and spontaneous ventilation even with small diameter tubes and routine inflation of the cuff is not recommended unless PPV is required (1, 9). Should PPV be required, pre-hospital HCPs may be limited to self-inflating bag-valve (BVM) devices rather than ventilators, which can compensate for tube leaks caused by an incomplete airway seal (1). Therefore, the second aim of this study is to establish whether it is possible to support PPV and compensate for tidal volume (TV) loss, using devices such as BVM, *via* CTT tubes in dogs.

The third aim is to find the best performing tube of those tested, and the fourth is to explain the findings using images and measurements of the airway.

The authors therefore hypothesize: (1) In dogs of similar body weight to MWD and Operational K9 breeds, the 10.0 and 8.0 mm tubes would have greater success at achieving an airway seal than the smaller commercial CTT tubes; (2) TV loss will be small enough to deliver a minimum effective TV using a BVM for all tubes and (3) The larger 10.0 and 8.0 tubes will perform better in both tests than the commercial CTT tubes.

## Materials and methods

Animal ethics approval was granted from the University of Queensland Animal Ethics Committee, approval number ANRFA/159/20. Ten canine cadavers were reused from previous teaching labs and were selected based on the body weight range of common MWD breeds (20–40 kg) (10, 11).

The commercial CTT kits (Portex^®^, Melker^®^, and H&H^®^) were included for testing based on their popularity in the literature and availability. A standard orotracheal tube and a standard tracheostomy tube were also included as these tubes are commonly available in prehospital and acute care settings.

Therefore, the five tubes tested were:

- Portex^®^ PCK 6.0 mm I.D (PCK).^1^
- Melker^®^ cricothyrotomy tube 5.0 mm I.D (Melker).^2^
- H&H^®^ cricothyrotomy tube 6.0 mm I.D (H&H).^3^
- Generic, unbranded polyvinylchloride endotracheal tube 8.0 mm I.D (8.0 mm).
- Portex^®^ cuffed blueline ultra^®^ suctionaid 10.0 mm I.D (10.0 mm).^4^

The cuff of each tube was inflated to 20 cm H_2_O and the external diameter was measured using digital calipers before tube insertion.

A cricothyrotomy was performed on each cadaver and each tube was tested using the same incision and in the same order. After tube insertion, the tube cuff was inflated to 20 cm H_2_O using the AG Cuffill™^5^ device with the inflation volume recorded. The AG Cuffill™ syringe has been validated for accuracy in a previous veterinary study (12) and was used to measure all cuff volumes and pressures. Subsequently, the tube was connected to an ICU ventilator^6^), which had passed a leak and circuit compliance test before data collection. The ICU ventilator was used to ensure consistent and identical pressure-controlled breaths were delivered for every test.

### Cuff sealing using the minimum occlusive volume technique

In the context of this study, a leak was defined as an audible air leak during a positive pressure breath by the ventilator. All leak assessments were performed by the same researcher (SH), at the same locations of the CTT site and mouth. Dogs with audible leaks underwent a MOV cuff inflation, where the tube cuff was gradually filled with air during a ventilator-delivered breath until leaks were no longer audible up to the target peak inspiratory pressure (PIP) of 15 cm H_2_O, unless maximum cuff inflation (>99 cm H_2_O) was reached at a lower PIP. All additional inflation volumes and pressures over the initial 20 cm H_2_O IC pressure were recorded. The maximum pressure reading from the cuff inflation device was 99 cm H_2_O and IC pressures above this were recorded as over pressure (OP). For this study, a successful airway seal was defined as absence of audible air leak at a PIP of 15 cm H_2_O and a cuff pressure of 48 cm H_2_O and below.

### Tidal volume loss assessment as a measure of leak volume

For this study, the ventilator leak compensation function was enabled to accurately measure the tidal volume loss during the inspiratory cycle of ventilation. The function corrects for air leak 200 times per second and this model ventilator has been reported to have the most accurate leak compensation function amongst several ICU ventilators investigated in its class (13). Ventilation was set at 20 breaths per minute with an inspiratory: expiratory ratio of 1:2 and 50% ramp. The PIPs of 5 and 10 cm H_2_O were measured to ascertain lower PIP limits in case the magnitude of TV loss at the target of 15 cm H_2_O was too large. After connecting the ventilator to the CTT tube, ventilation continued until stabilization of mean airway pressure. At least two identical leak volumes were obtained before recording this as the final leak volume. Cadavers 1–3 were a pilot group, used primarily to assess the feasibility of the experiment. With this group, tube cuff to trachea interactions were visualized with endoscopy and airway sealing using the MOV method of cuff inflation. Subsequent cadavers (4–10) had TV leak measured up to a PIP of 15 cm H_2_O with an IC pressure fixed at 20 cm H_2_O, in addition to assessment of airway sealing using the MOV method.

Individual tidal volumes were calculated as 8 ml/kg as this volume was able to produce clinically acceptable ventilation in a study of dogs without the use of PEEP (14). The calculated TV was added to the leak volume for each test to ascertain the total volume required to deliver a breath over 1 s. This total volume was compared against an average breath delivered by a BVM device (785 ml) (15). If the combined tidal volume and leak volume was < 785 ml, it was considered possible to deliver a breath with a PIP of 15 cm H_2_O using a BVM device.

### Endoscopy

Video endoscopy was performed during data collection for all tubes in the pilot group, consisting of cadavers 1–3. Endoscopy was performed by the same individual, who was a board-certified specialist in small animal internal medicine. Images were captured to document interactions between tube cuffs and the trachea during ventilation. A selection of images that demonstrate key findings have been included in the results.

### Airway measurements

**Figure 1:**
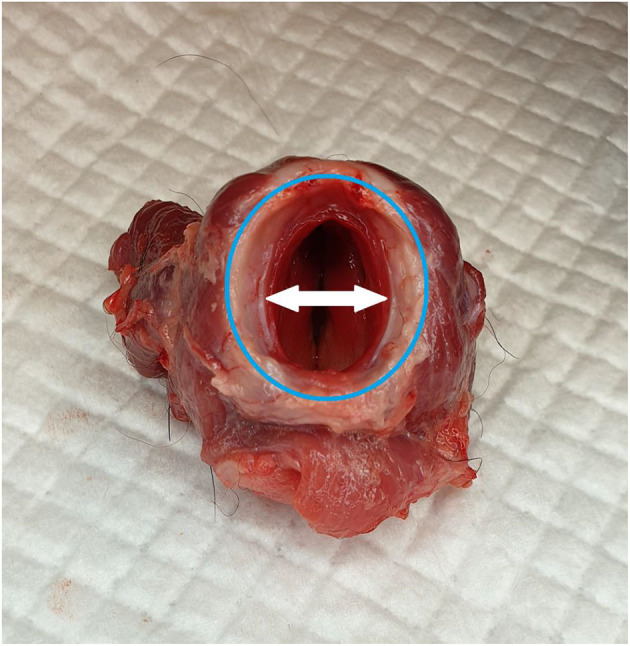
Caudal view of the larynx, with the ventral aspect toward the top of the image. The shape of the caudal cricoid is more circular when compared to the ovoid trachea (highlighted by the blue ring). The internal diameter at the narrowest point of the cricoid ring is smaller laterally, which is opposite to the trachea (white arrows).

The airways of all cadavers were dissected after testing the tubes. For the cricoid ring measurements, the lumen was measured at the narrowest point of caudal cricoid cartilage in the lateral and dorsoventral planes using digital calipers (Arrows in Figure 1 demonstrate the lateral measurement). The trachea was removed, and the internal dimensions (lateral and dorsoventral) were measured at 5 and 10 cm distal to the larynx. To calculate the cricoid and tracheal lateral to dorsoventral (DV) ratios, the lateral measurement was divided by the DV measurement. Observations made on endoscopy were demonstrated grossly by placing the tubes in the dissected airway and inflating them to the initial cuff pressure of 20 cm H_2_O.

### Statistical analysis

Data were summarized in accordance with their distribution and type, with normally distributed data as mean (SD), non-normal data as median (range) and where appropriate, categorical/binary data as proportion (%).

To model the success of the five CTT tube types creating a functional seal against the tracheal mucosa at an inspiratory pressure of 15 cm H_2_O for the ten cadavers (1–10), a logistic regression model with Bayesian inference was used to yield estimators for all tube types, with normal and uniform non-informative prior distributions trialed. An adaptive Metropolis-Hastings (MH) Markov chain Monte Carlo (MCMC) process with 100,000 iterations and a burn-in of 5,000 was used. For the second research question, to model the leak volume (ml) from the five tubes with the cuff inflated to 20 cm H_2_O at an inspiratory pressure of 15 cm H_2_O for the seven cadavers (4–10), a frequentist linear regression model was used. For both models, the explanatory variables trialed were the tube type and a measure of airway circularity as defined by the composite variables of cricoid and tracheal ratios of lateral dimensions divided by dorsoventral dimensions. A random intercept and coefficient was also trialed for individual variation between dogs in leak volumes and differing effect of tube type by dog, respectively. A forward stepwise approach was used for model selection, with posterior probabilities and Bayes Factors used for the Bayesian logistic regression model and likelihood ratio tests for the linear regression model. The baseline tube for both models was the 8.0 mm, with all other tubes compared to the 8.0 mm.

The significance level was set at 0.05 and all analyses were conducted in Stata version 16.1.

## Results

### Study population descriptives

The study population of canine cadavers consisted of two Greyhounds (cadavers 2 and 3) six Mastiff crosses (cadavers 1, 4, 5, 8, 9, and 10), a Bull terrier cross (cadaver 6) and a Rottweiler (cadaver 7). There were five males, five females and the median weight was 27.2 kg (IQR: 26.3 to 29.7). The median (IQR) internal airway dimensions at the caudal cricoid were 22.3 (21.0–23.5) mm dorsoventrally (DV) and 17.0 (15.8–17.5) mm laterally. The median tracheal dimensions 5 cm distal to the larynx were 18.6 (17.4–18.9) mm DV and 22.1 (20.4–24.4) mm laterally, and 10 cm distal to the larynx were 18.0 (16.6–18.7) mm DV and 21.8 (19.9–23.0) mm laterally. The mean (SD) tracheal ratio for measurements taken 10 cm distal to the larynx (lateral:DV) was 1.21 (0.14). All tube types were trialed in all cadavers, and there were no missing values. Further details regarding cadaver characteristics, airway dimensions and TV leak volumes can be found in Supplementary Table 1.

#### Cuff sealing using the minimum occlusive volume technique

**Figure 2:**
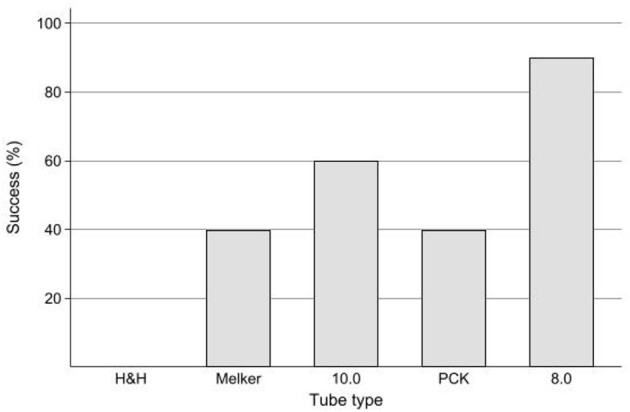
Bar chart of successful airway seal (%) using the minimum occlusion volume technique with intra-cuff pressure ≤ 48 cm H_2_O in five tubes used for cricothyrotomy in ten cadaver dogs. Success (%) describes a successful airway seal between tube cuff and trachea as defined as absence of audible air leak at a PIP of 15 cm H_2_O and a cuff pressure of ≤ 48 cm H_2_O. Tube type definitions: PCK–Portex^®^ PCK 6.0 mm I.D.; Melker–Melker^®^ cricothyrotomy tube 5.0 mm I.D.; H&H–H&H^®^ cricothyrotomy tube 6.0 mm I.D.; 8.0 mm–Generic, unbranded polyvinylchloride endotracheal tube 8.0 mm I.D.; 10.0 mm–Portex^®^ cuffed blueline ultra^®^ suctionaid 10.0 mm I.D.

The measurements and descriptions of the tubes used in the study are shown in Table 1. Leaks were rarely audible at the level of the CTT incision due to soft tissue covering the site. Airway sealing was successful in 22/50 (44%) tests. The tubes with the highest success rates were the 8.0 mm tube and 10 mm at 90 and 60%, respectively. The Melker and PCK tubes had equal success at 40%, and the H&H tube failed all tests (Figure 2). It was rare that a seal was maintained with the lowest cuff pressure of 20 cm H_2_O at a PIP of 15 cm H_2_O with only two instances from the Portex (cadavers 7 and 10), two from the Melker (4 and 7), three from the 8.0 mm (4, 5, 9) and one from the 10.0 mm (7). The raw data for cuff pressures and volumes required to seal the airway can be found in the Supplementary Table 2.

**Table 1:** Tube characteristics for the five tubes placed *via* cricothyrotomy in 10 cadaver dogs.

**Tube design**	**Measured cuff diameter after inflation to 20 cm H_2_O IC pressure (mm)**	**Inner/outer tube diameter (mm)**	**Labeled cuff diameter (mm)**	**Labeled length (mm)**	**Measured length (mm)**	**Manufacturer recommended cuff inflation instructions**	**Measured cuff volume at 20 cm H_2_O (ml) with tube in airway. Mean (SD)**
PCK	26.0	6.0/9.2	23.0	90	94	MOV technique	7.5 (0.85)
Melker	21.6	5.0/7.2	22.0–29.0	90	95	8–10 ml	4.5 (0.83)
H&H	16.7	6.0/8.0	–	–	66	–	3.4 (0.63)
8.0 mm	26.4	8.0/10.7	–	–	–	–	Med 4.7 (1.8)
10.0 mm	30.5	10.0/14.0	30.0	87.5	97	20–30 cm H_2_O	Med 4 (1)

PCK, Portex^®^ PCK; Melker, Melker^®^ cricothyrotomy tube; H&H, H&H^®^ cricothyrotomy tube; 8.0 mm, Generic, unbranded polyvinylchloride endotracheal tube; 10.0 mm, Portex^®^ cuffed blueline ultra^®^ suctionaid.

##### Success of airway sealing and tracheal width to height ratio

**Figure 3:**
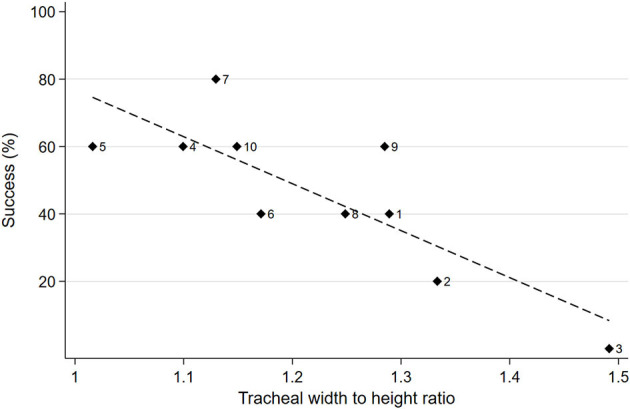
Scatterplot of percent of successful airway sealing for all tubes by lateral to dorsoventral tracheal ratio with cadaver dog ID next to diamond markers, with line of best fit.

There was a significant negative correlation of 0.82 between the tracheal ratio and the success of airway sealing for all cadavers (*P* = 0.004). The dogs with a higher lateral to dorsoventral tracheal ratio demonstrated a grossly flatter, ovoid trachea rather than a circular cross section. These cadavers, such as cadaver 3, had a lower success rate than dogs with more circular tracheal cross section, that is, a tracheal ratio closer to 1 such as cadavers 4, 5, and 7 (Figure 3).

##### Model for association between successful airway seal, tube type and tracheal lateral to dorsoventral ratio

Bayesian inference with a logistic regression model was used to estimate the multivariate adjusted odds ratio (OR) of success for airway sealing in all cadaver dogs, with the tracheal ratio centered at the mean and multiplied by 10 to aid in interpretation of the coefficients (Table 2). The predicted probabilities are for an individual dog with a tracheal ratio at the mean. Non-informative prior distributions were used; for the tube and tracheal ratio parameters this was uniform [−10, 10] and for the intercept this was normal (0, 10). The random effect was removed due to lack of contribution to the model.

**Table 2:** Bayesian logistic regression model for estimating of odds ratio of successful airway sealing in ten cadaver dogs with different cricothyroid tubes placed.

	**Odds ratio**	**Equal-tailed 95% CRI**	**Posterior probability**
Constant	25.39	(2.29, 123.10)	0.002
Tube			
8.0 mm (baseline)	–	–	–
H&H	0.0015	(0.000050, 0.011)	< 0.0001
Melker	0.076	(0.0023, 0.39)	0.005
10.0 mm	0.26	(0.0085, 1.39)	0.09
PCK	0.079	(0.0022, 0.41)	0.006
Centered tracheal ratio (^*^10)	0.32	(0.12, 0.62)	0.0004

CRI, credible interval; Tube type definitions: PCK, Portex^®^ PCK 6.0 mm I.D.; Melker, Melker^®^ cricothyrotomy tube 5.0 mm ID; H&H, H&H^®^ cricothyrotomy tube 6.0 mm I.D.; 8.0 mm, Generic, unbranded polyvinylchloride endotracheal tube 8.0 mm I.D.; 10.0 mm, Portex^®^ cuffed blueline ultra^®^ suctionaid 10.0 mm I.D. (^*^10): tracheal ratio centered at the mean and multiplied by 10 to aid in interpretation of the coefficients. Probability are posterior probabilities for the parameters being < 0, except for the constant which is >0. This is similar to the one-sided frequentist likelihood ratio test P-value, and so these values were multiplied by 2.

The baseline tube was the 8.0 mm with a predicted probability of successful airway seal of 90.7% (95% CRI: 69.6–99.2) for an individual dog with a tracheal ratio at the mean. The H&H tube had a statistically significantly reduced odds of success compared to the baseline 8.0 mm tube, with the odds estimated to be 99.8% lower (95% CRI: 98.9–99.9; posterior probability < 0.0001) and the estimated mean probability of success being 1.4% (95% CRI: 0.03–8.8). The Melker and PCK tubes also had reduced odds of success in comparison to the 8.0 mm tube with an estimated mean probability of success of 35.1% (95% CRI: 8.5–69.7) and 35.0% (95% CRI: 8.5–69.3), respectively. For the 10.0 tube the odds of airway seal was 74% lower than the 8.0 mm tube and this was not a statistically significant reduction (95% CRI: 99.2% lower to 39.1% higher) (Table 2); the estimated mean probability of success was 60.8% (95% CRI: 26.3–90.1%).

There was a significant association between success and tracheal lateral to DV ratio (*P* = 0.0004) and for each 0.1 unit increase in tracheal ratio, there was a 68.3% (95% CRI: 38.5–87.8) lower odds of successful airway seal, for a given tube type.

The model was compared to a logistic regression model using a frequentist approach with the H&H tube removed given it is a perfect predictor; the results were substantively similar and so the chosen prior distributions appeared to have little effect.

##### Tidal volume leak

**Figure 4:**
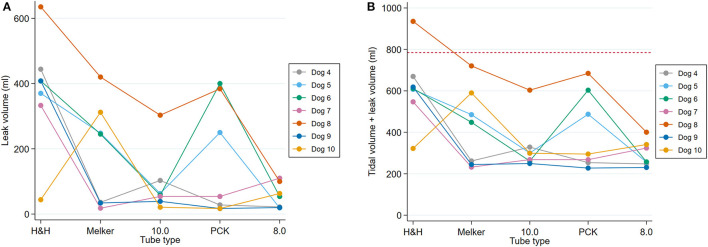
Line plot of **(A)** tidal volume leak (ml) at 15 cm H_2_O PIP and 20 cm H_2_O IC pressure by tube type. **(B)** Sum of calculated tidal volume and leak volume with red dashed line at 785 ml indicating potential for breath delivery using BVM (dogs 4–10).

At 15 cm H_2_O PIP and 20 cm H_2_O IC pressure for dogs 4–10, the median leak volume was 63 ml (IQR: 34–333) with a range of 17–635 ml. Figure 4A shows leak volumes for each tube in each dog. Overall, the 8.0 mm and PCK has the lowest median TV leak volumes of 54 ml (IQR: 20–100, range: 19–110) and 54 ml (IQR: 17–384, range 17–400), respectively. For the remaining tubes, median TV leak volumes in ascending order were the 10.0 mm, Mekler and H&H with 58 ml (IQR: 39–103, range 21–303) 245 ml (IQR: 34–312, range: 18–420) and 408 ml (IQR: 333–444, range: 44–635), respectively. The sum of the TV leak and individual dog's TV is shown in Figure 4B and graphically compared to the average BVM breath volume (785 ml). This volume was exceeded in only one dog, cadaver 8 using the H&H tube.

##### Model for association between tidal leak volume and tube type

A random-coefficient multivariate linear regression model was used to estimate the association between tidal leak volume and tube type, with cricoid and tracheal ratios also trialed as a measure of airway circularity (Table 3). When trialed, the tracheal ratio did not contribute significantly to the model. The model showed very strong evidence of a difference in mean tidal leak volume between tubes (*P* < 0.001), after adjusting for the lateral to DV cricoid ratio. However, cadaver 8 was an outlier and, as shown in Table 3, the cricoid ratio was no longer significant when it was removed (*P* = 0.14).

**Table 3:** Linear regression model estimates, with and without dog ID 8.

	**Model with ID 8**			**Model without ID 8**		
	**Regression coefficient**	**95% CI**	***P*** **-value**	**Regression coefficient**	**95% CI**	***P*** **-value**
**Tube**						
Constant (8.0 mm)	55.4	(39.9, 71.0)	< 0.001	50.5	(28.6, 72.5)	< 0.001
H&H	322.0	(193.4, 450.6)	< 0.001	286.5	(164.6, 408.4)	< 0.001
Melker	132.1	(18.5, 245.8)	0.02	100.8	(−11.0, 212.7)	0.08
10.0	36.1	(−28.2, 100.5)	0.27	8.3	(−27.3, 43.9)	0.65
PCK	108.9	(−16.9, 234.6)	0.09	79.7	(−51.7, 211.0)	0.24
Centered cricothyroid ratio (^*^10)	52.8	(26.4, 79.1)	< 0.001	22.7	(−7.2, 52.6)	0.14

The random intercept and coefficient both contributed significantly to the model [χ^2^ (1) =5.9, *P* = 0.02 and χ^2^ (4) = 9.5, *P* = 0.05, respectively].

**Figure 5:**
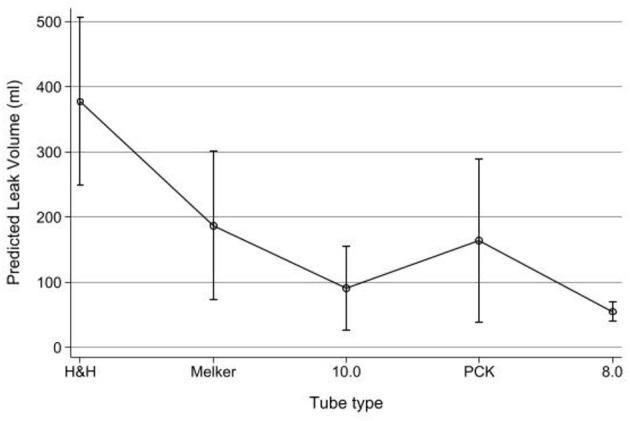
Predicted tidal volume leak (ml) by tube type (dogs 4–10). Point estimates, whiskers = 95% CI.

The tidal volume leak for all tubes are compared in Figure 5. The highest mean leak volume was 377.4 ml (95% CI: 248.6–506.3) for the H&H tube. The Melker and PCK tubes had the next highest mean leak volumes with 187.6 ml (95% CI: 73.6–301.5) and 164.3 ml (95% CI: 38.3–290.3), respectively. The lowest mean leak volumes were recorded for the 8.0 mm tube with 55.4 ml (95% CI: 39.9–71.0) and 10.0 tube with 91.6 ml (95% CI: 26.8–156.4).

### Observations of tube cuff to trachea interactions

**Figure 6:**
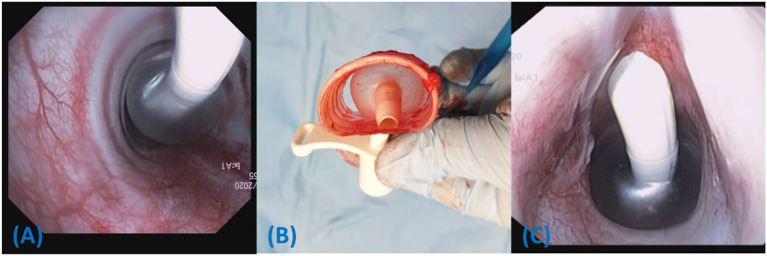
Ventral is toward the top of the image. **(A)** The Melker tube is shown *in situ* in cadaver 2. Air can escape lateral to the cuff, particularly in tracheas with higher lateral to dorsoventral ratios. **(B)** The Melker tube is shown in a dissected portion of the trachea from cadaver 3. Contact on the dorsal and ventral trachea occurs first when inflated to a pressure of 20 cm H_2_O. **(C)** Hyperinflated tube cuff from Melker tube, pulled proximally to lodge at the caudal cricoid ring in Cadaver 1. An effective seal can be observed as the spherical cuff conforms to the circular boundary of the caudal cricoid ring.

The anatomy of the caudal larynx is shown in Figure 1. Toward the caudal aspect of the cricoid ring, the lateral dimensions increase creating a more circular shape. In cadaver dog 1, this was observed to have an effect on airway sealing as tube cuffs with circular cross sections could conform more closely to the circular shape of the caudal cricoid when pulled proximally (Figure 6C). Due to the overall large size and ovoid shape of the trachea in the cadavers tested, the commercial CTT tube cuffs sometimes had difficulty reaching the lateral aspects of the trachea and would allow air leak at IC pressures 20 cm H_2_O (Figures 6A, B).

**Figure 7:**
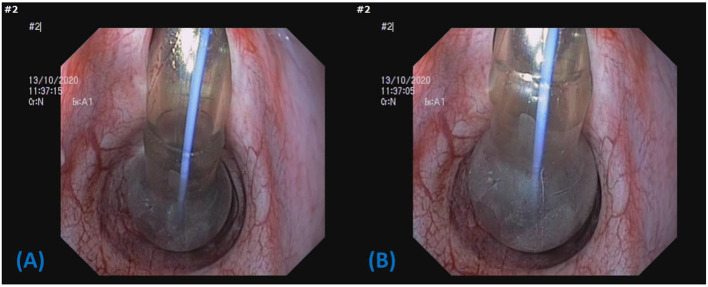
Image depicting the low volume, high pressure cuff of the H&H cricothyrotomy tube in cadaver dog 2. **(A)** Inflated to 20 cm H_2_O, **(B)** inflated as much as possible >99 cm H_2_O. The tube cuff contacts the airway at a single point and the IC pressure is unlikely to be transferred to the mucosa.

**Figure 8:**
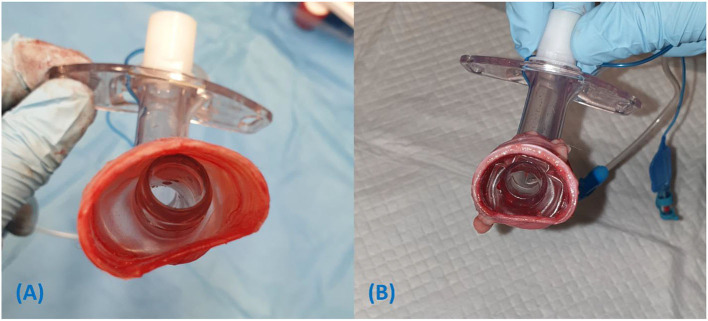
**(A)** The tube cuff of the 10.0 mm tracheostomy tube inflated to 20 cm H_2_O conforms to the ovoid shape of the trachea in cadaver dog 3. **(B)** Folding of the 10.0 mm tube cuff within the relatively small trachea of cadaver dog 9, allowing channels for air leak. Cadaver 9 had air leak with the larger 10.0 mm tube but not the smaller 8.0 mm tube.

The low volume, high pressure cuff of the H&H tube was too small to produce an effective seal at IC pressures 20 cm H_2_O (Figure 7A) and had difficulty contacting the tracheal mucosa on more than one aspect, even when hyperinflated (Figure 7B). Conversely, the 10.0 tube cuff had no difficulty conforming to even the largest tracheas (Figure 8A). However, it is possible that such a large cuff can fold when placed in smaller tracheas, potentially allowing small air leaks (Figure 8B).

## Discussion

This study sought to assess the effectiveness of airway sealing using various CTT tubes and to determine if PPV could be delivered despite TV losses using cadaver dogs as an anatomical model. The results suggest the commercial CTT tubes tested are unlikely to provide a safe functional airway seal using the MOV technique, however, larger standard endotracheal tubes may perform better. Based on the cadaver TVs and the leak volumes during PPV, it was determined that it was theoretically possible to deliver an effective pressure-controlled breath using a BVM.

### Sealing using the minimum occlusive volume technique

The H&H tube had the smallest cuff diameter, and as predicted, was the worst performing tube in terms of providing an airway seal using the MOV technique as it failed to provide an airway seal in all cadaver dogs.

Interestingly, the measured and manufacturer indicated cuff diameter was not always an accurate predictor of seal effectiveness when compared to tracheal dimensions, particularly when inflated to the minimum target pressure of 20 cm H_2_O. For example, the Melker's cuff diameter is expected to reach between 22 and 29 mm, which would exceed the majority of tracheal measurements in this study. Based on the anatomical observations in cadaver dogs 1–3, it appeared that due to the ovoid shape of the trachea, the cuff contacted the dorsal and ventral trachea first. The pressure on these contact points would contribute the most to the rise in IC pressure, possibly allowing it to reach 20 cm H_2_O before the cuff expands to its intended diameter. As a result, there is poor cuff sealing laterally, allowing air to escape around the cuff (Figures 6A, B). This concept is supported by the strong negative correlation of 0.82 between lateral to dorsoventral tracheal ratio and successful seal where the chance of a successful airway seal diminishes, with relatively larger lateral measurements (Figure 3).

The starting intracuff (IC) pressure of 20 cm H_2_O was chosen with the intention to incrementally increase inflation, consistent with similar studies assessing leakage past endotracheal cuffs (16). This would also represent the “worst-case scenario” for sealing capability by using the minimum recommended pressure. It was observed that when inflating a round cuff in an ovoid trachea, deformation of the trachea in the dorsoventral plane is required for the cuff to achieve maximum diameter, hence the number of OP readings required to achieve a seal using the MOV method of cuff inflation with the smaller tubes. It is important to note that these contact points would be exposed to a much higher pressure than the lateral aspects of the trachea and would be most prone to mucosal damage. This issue was not as pronounced when using larger tubes with larger diameter tube cuffs. For example, the character of the size 10.0 tube cuff is demonstrated in Figure 8A, where the cuff is shown to conform to the shape of the trachea, filling the lateral aspects to form a seal without deforming the trachea in the DV plane.

There is risk for tracheal mucosal necrosis and/or rupture when tube cuffs are maximally inflated. However, IC pressures are dependent upon “cuff diameter, thickness, compliance, shape trachea size and cuff position in the trachea” (7) and IC pressures only reflect the pressure exerted upon the tracheal mucosa in high volume, low pressure cuffs (6). With low volume, high pressure cuffs (such as the H&H) or even high volume cuffs with small diameters, the addition of a small volume can result in a marked increase in pressure. However, this pressure would not necessarily be transferred to the tracheal mucosa unless there are at least two contact points to oppose the force through the tube cuff. As depicted in Figures 7A, B, in some cases the cuff may only contact the mucosa on one surface or have no contact at all, even when hyperinflated. The disparate diameters between the cuff and the trachea may result in a reduced risk of pressure necrosis. Hence, small cuffs have the potential to be inflated to high pressures safely when placed in relatively large tracheas. Due to the variability in tracheal anatomy and cuff compliance, the average cuff inflation volumes observed in this study cannot be used to predict a safe and effective volume for each tube.

An interesting observation noted in this study, was the 8.0 mm tube was better at creating a successful seal compared with the 10.0 tube which had the largest diameter cuff (90 vs. 60% successful seal, respectively), although this was not statistically significant. This seems counterintuitive, however, with smaller tracheas, a large tube cuff folds in on itself and results in an uneven contact surface between the cuff and tracheal mucosa. Air and fluid have the potential to egress and ingress through tracts made by longitudinal folds in the cuff. This is demonstrated in Figure 8B and supported by Hwang et al. (16) who found size 7.0 mm I.D. tubes formed a better seal against fluid and air leakage than larger sized 7.5 and 8.0 mm I.D. tubes in the same experimental tracheal model. The results from the current study also suggest that larger tubes and cuffs do not necessarily correlate with a better airway seal. Furthermore, using the MOV has the potential to result in dangerously high IC pressures, with an unpredictable and sometimes inconsequential reduction in perceived audible air leak as long as the folds remain.

Another potential reason for the superior performance of the 8.0 mm is the length of the tube. Although efforts were made to insert the tube to 10 cm, this was based on markings on the tube, whereas all other tubes were shorter and had a physical barrier to further advancement consisting of a collar used to secure the tube to the neck. The trachea tends to become slightly narrower and more circular toward the thoracic inlet, which may provide a better airway seal around a smaller cuff (17–19). However, it is unlikely the tube consistently migrated very far in the majority of cadavers. Even if this did occur, this would only support the 8.0 mm tube's superior performance when applied practically, as it has the potential to be advanced further if necessary.

Leakage of fluid and subsequent microaspiration has been reported to occur even in controlled situations using appropriate tube sizes (16, 20). Hence the requirement for a perfect seal is questionable, particularly when the primary goal of a CTT intervention is to provide immediate oxygen and ventilation before the patient succumbs to hypoxia. The glottis is no less protected than when using mask ventilation techniques, which is the primary recommendation for ventilation in most prehospital trauma patients without airway obstruction (1). Subsequently, the goal is to have the patient conscious as soon as possible to allow for airway protective reflexes to return.

### Tidal volume leak

With regards to TV leak, the hypotheses were supported, with all but one of 35 tests performed able to support the delivery of a calculated TV based on weight and a standard BVM breath through a CTT tube under the parameters set in the experiment. Additionally, the three commercial CTT tubes with the smallest tube cuffs had the largest leak volumes.

An unexpected finding was the 8.0 mm standard endotracheal tube was the best performing tube with the lowest mean TV leak despite having a smaller diameter cuff than the 10.0, although this was not significant in this study at the 5% level. This is postulated to be due to tube cuff characteristics, creating a tract for air to escape, or the tube length as explained above. The subjective assessment for audible leaks and the objective measurement of tidal volume leak both support a relative performance advantage of the 8.0 mm tube over the 10.0 mm despite having a smaller cuff diameter. A larger sample size may have resulted in a significant difference in performance between these tubes.

A further interesting finding was that reduced cricoid lateral to DV ratios were significantly correlated with decreased TV loss, although this could be a spurious finding due to the outlier as explained in the results. The cricoid ring did not interact with the tube cuff due to its proximal location, however, a narrow cricoid width could conceivably explain reduced leak volumes, due to less space around the tube for air to escape. This was found to be the narrowest part of the proximal airway on average and could be a “second line” in restricting air leak. To the authors' knowledge, this characteristic of the internal cricoid ring has not yet been described in previous studies assessing the measurements of canine airways (17–19). Only a small amount of air is required to produce an audible leak, which may explain why the tracheal ratio was not associated with leak volume.

A possible limitation of this study was the use of canine cadavers. It was anticipated that lung and chest wall compliance may not mirror that expected in live specimens. Therefore, the intention was to use the cadaver airways as an *in vitro* anatomical model. Hence, all tests were performed under a pressure control mode of ventilation to ensure each breath was delivered consistently. The focus was to establish the leak volume alone, with the cadaver's measured tidal volumes ignored and substituted *post-hoc* with one calculated from bodyweight. In one study assessing volume controlled ventilation in dogs without PEEP, PIPs of 10 cm H_2_O and 14 H_2_O resulted when TVs of 8 and 15 ml/kg were delivered, respectively (14). A target PIP of 15 cm H_2_O was chosen as it would ensure the majority of cases requiring PPV receive adequate ventilation. Further, it gives some margin for reduced pulmonary compliance due to injury. There is little information in the literature regarding recommended pressure settings for mechanical ventilation as volume-control is often favored for lung protection and the resultant PIP would be dependent upon lung compliance (21).

Another possible limitation from using cadaver airways is that repeated cuff inflation in the same area may have led to tissue fatigue, reducing the efficacy of airway sealing in subsequent tests.

Unsurprisingly, the H&H tube (6.0 I.D., 16.7 mm cuff diameter) had a significantly greater TV leak than the 8.0 mm and 10.0 mm tubes and had the highest mean leak volume of all tubes. The greatest TV leak from the H&H tube was 635 ml at 15 cm H_2_O PIP (cuff inflated to 20 cm H_2_O) in cadaver 8. However, during maximum inflation of the tube cuff to >99 cm H_2_O, the TV loss decreased to 125 ml, which would be much easier to accommodate when delivering a breath. The act of inflating the cuff has a great impact on decreasing the leak around tubes and is of benefit for more effective ventilation (9). Based on our endoscopic examination, this would unlikely cause complications due to limited contact of the H&H cuff with the mucosa. However, continuing to inflate the cuffs from the other tubes can potentially give the high volume, low pressure cuffs, characteristics of a low volume, high pressure cuff and lead to complications (22). Considering there was only one failure for the combined leak and tidal volume to fall below 785 ml at an IC pressure of 20 cm H_2_O, the risk of potentially inflicting tracheal mucosal damage with high cuff pressures must be weighed against the very small chance of delivering an inadequate breath. Therefore, it is reasonable to target an IC pressure of 20 cm H_2_O at the commencement of PPV if possible.

### Theoretical clinical applications based on this data

Standard self-inflating adult BVM devices have a volume of 1,500 ml. Nurses in one study produced an average TV of 785 ml when delivering breaths during a simulation (15) and emergency service providers achieved a median of 982 ml in another study (23). A more conservative minimum volume was used in this study. From the 35 tests performed at a cuff pressure of 20 cm H_2_O, the combined leak volume and tidal volume exceeded the limit in one dog with the H&H tube. This of course, would not have failed had the more generous median volume of 982 ml been adopted. Again, the most conservative parameters were chosen with an inspiratory time of 1 s to allow the longest duration of time for tidal volume leak to occur (14). With low TV, respiratory rates may need to be increased to meet minute ventilation requirements. Shorter inspiratory times than 1 s with the same pressure targets used in this experiment will result in less leak and has the potential to deliver markedly larger TVs.

Tidal volumes < 8 ml/kg may also be effective. This volume was selected as the lowest effective TV from a previous study. However, those dogs were positioned in dorsal recumbency while ventilated on 100% FiO_2_. As this position reduces efficacy of gas exchange and a high FiO_2_ predisposes to atelectasis, it is reasonable to assume a patient managed in sternal recumbency would achieve superior ventilation characteristics, and therefore a reduced TV may be possible. In humans, a tidal volume of 6 ml/kg, has the ability to provide lung-protective ventilation in ARDS patients and was sufficient to maintain effective ventilation following anesthesia (21, 24). Nevertheless, based on the average TV losses and patient size, ventilation is likely possible using CTT tubes and a BVM in MWD and Operational K9 breeds. Healthcare providers may even consider trying to reduce average TVs when using a standard BVM as they may exceed patient requirements, causing lung injury irrespective of tube type.

One experimental study noted IC pressures dropped below 20 cm H_2_O in 31% and 10 cm H_2_O in 20% of tubes when cuff pressure control maneuvers were performed by experienced ICU nurses (25). It is also possible this occurred during testing in the current study, resulting in more leak than expected. In practice, the majority of cuff inflations exceed recommended pressures (12, 26). Hence, the conditions set in this experiment likely represent a worse-case scenario by accurately providing a low starting point. In reality, there is potential to achieve markedly less TV leakage than our results indicate. All leak volumes were also recorded during MOV maneuvers in our study and demonstrate marked decreases in leak with cuff pressures >20 cm H_2_O.

In instances where CTT is performed due to upper airway obstruction, the obstruction itself would restrict airflow through the upper airway, therefore reducing TV leakage when PPV is applied. Numerous live-animal studies employ this concept to facilitate effective ventilation through uncuffed tubes and even intravenous cannulas (27–30). If excessive leak is a concern, the HCP can consider placing an examination glove over the muzzle or simply holding it shut to improve the effectiveness of IPPV.

### Airway anatomy

Many findings in this study are explained by the airway anatomy of the dog. A limitation to this study is that tests were not performed in German Shepherd dogs or Belgian Malinois, the latter of which has been the most common MWD breed for the past few decades (31). However, cadavers of similar size were sourced based on availability. Airway conformation is likely a critical factor in enabling tube cuff sealing and PPV. To the authors knowledge, there are no studies assessing airway dimensions in the Belgian Malinois. However, one study assessed tracheal dimensions in the German Shepherd dog using computed tomography (19). Their average body weights were within 1 kg of our cadavers and average tracheal dimensions within 2 mm. Considering the comparable measurements, the range of cadavers used could be considered a reasonable substitute for this proof-of-concept study. Interestingly, body weight did not appear correlated with tracheal dimensions in our study sample, with the 27 kg Greyhound having larger measurements than the 37 kg Mastiff. It is likely that tracheal dimensions and ratios are breed-dependent.

The potential for disparate diameters between tracheas and CTT tube cuffs has also been noted in human healthcare (32) and findings from this study is likely to generate further discussion in this field. The average tracheal dimensions (lateral and dorsoventral) in men aged 20–29 years were 18.7 × 19.3 mm, with dorsoventral measurements consistently greater in humans across all age ranges (33). In contrast, lateral tracheal measurements were consistently greater than dorsoventral measurements in dogs from this study. The largest tracheal dimensions in humans measured 27 mm in one study, which is similar to the largest tracheal measurement in this study of 27.5 mm. Some observations from this study with regards to small tube cuffs have the potential for application in humans. The measurements of proximal human tracheas indicate a rounded cross-section with an almost identical ratio of DV to lateral measurements in the 20–29 year age group of men being 0.97. This study population of dogs had a ratio of 1.2, which supports previous studies demonstrating a greater lateral to DV ratio of the extrathoracic trachea (18, 19). This also explains the ineffective airway seal and greater leak volumes observed, where air escapes around the side of the cuff of the smaller tubes. Perhaps, if these tubes were longer, cuff sealing of the airway may be more effective in the thoracic trachea where the lateral to DV ratios are closer to 1. Given the poor results of the H&H tube, the effectiveness of its design should be questioned, in particular, the low volume, high pressure cuff.

The caudal cricoid cartilage and larynx, grossly appeared circular (Figure 1), compared with the ovoid trachea. If tubes are pulled more proximally to the caudal larynx, rather than inserted to ~10 cm (as done in the present study), a rounded tube cuff would likely conform better to the airway, reducing TV leakage (Figure 6C). This would be difficult to employ in the field, as the cuff diameter cannot be monitored and tube cuffs such as the H&H would simply pass through the cricoid ring, dislodging the tube from the airway completely.

Larger studies in live MWD and Operational K9 breeds with the addition of arterial blood gas analysis are required to verify the results from this proof-of-concept study. However, this may not be practical or ethical.

## Conclusions

In conclusion, the standard 8.0 mm endotracheal and 10.0 mm tracheostomy tubes performed better than the commercial CTT tubes in producing a safe and effective airway seal. An airtight seal was not safely obtained with the majority of commercial CTT tubes and the MOV technique of cuff inflation frequently resulted in dangerously high intra cuff pressures. However, despite the leaks, an effective breath is possible with a BVM using all tube types at the minimum cuff pressure of 20 cm H_2_O. The 8.0 mm endotracheal tube was the most appropriate for the current study's population of dogs with the least TV loss and greatest success at safely providing a functional airway seal using the MOV method of cuff inflation. Cuff to trachea interactions were more important in providing an air-tight seal and reducing leaks than simply selecting the largest diameter cuff, as evidenced by the 8.0 mm outperforming the 10.0 mm in both tests. Therefore, standard endotracheal tubes should be considered for prehospital CTT in working dogs if available. If human equipment is used, the limitations and optimal use conditions identified in this study can be considered.

## Data availability statement

The original contributions presented in the study are included in the article/Supplementary material, further inquiries can be directed to the corresponding author.

## Ethics statement

The animal study was reviewed and approved by the University of Queensland Animal Ethics Committee, approval number ANRFA/159/20.

## Author contributions

SH: conception, design of the study, data collection, and manuscript preparation. SH and MH: execution of the experiment and data recording. SH, MH, SP, CC, and WG: manuscript drafting and review. CC: results and statistical analysis. All authors contributed to critical revisions of the article and approved the submitted version.

## Footnotes

^1^Smiths Medical Australasia Macquarie Park, Macquarie Park, NSW, Australia.

^2^Cook Medical LLC, Bloomington, IN, United States.

^3^H&H medical corporation, Williamsburg, VA, United States.

^4^Smiths Medical Czech Republic a.s. Olomoucká 306, Hranice, Czechia.

^5^KRUUSE, Havretoften 4 DK-5550, Langeskov, Denmark.

^6^Coviden Puritan Bennett™ 840.
