# Supplementary material for: Pre-hospital emergency cricothyrotomy in dogs part 2: Airway sealing and ventilation using cricothyrotomy tubes
Source: Front Vet Sci. 2023 Feb 17;10:1129462. doi: 10.3389/fvets.2023.1129462 (PMC9981793; doi:10.3389/fvets.2023.1129462)
Supplement: Supplementary file 1 [file Table_1.DOCX]

**Table 1: Volume of leak (ml) at 15 cm H_2_O PIP and 20cm H_2_O IC pressure and airway dimensions of caudal cricoid and trachea at 10 cm distal to larynx by dog ID (dogs 4 to 10 only with leak shown)**

| **ID** | **Breed** | **e** | **Volume of leak**  **(ml)** | | **Cricothyroid width**  **(mm)** | **Cricothyroid height**  **(mm)** | **Tracheal width (mm)** | **Tracheal height (mm)** | **Tracheal Ratio (width /height)** |
| --- | --- | --- | --- | --- | --- | --- | --- | --- | --- |
| e |  |  | **Median (IQR)** | **Range** |  |  |  |  |  |
| **1** | Mastiff x | 23.0 | - | - | 13.8 | 21.6 | 21.4 | 16.6 | 1.29 |
| **2** | Grey-hound | 27.0 | - | - | 17.5 | 23.1 | 22.0 | 16.5 | 1.33 |
| **3** | Grey-hound | 27.4 | - | - | 18.6 | 23.5 | 26.4 | 17.7 | 1.49 |
| **4** | Mastiff x | 28.2 | 36  (28 – 103) | 22 to 444 | 17.4 | 24.4 | 19.9 | 18.1 | 1.10 |
| **5** | Mastiff x | 29.7 | 248  (63 – 250) | 19 to 370 | 15.8 | 20.2 | 18.8 | 18.5 | 1.02 |
| **6** | Bull terrier x | 25.4 | 245  (58 – 400) | 54 to 408 | 16.9 | 22.2 | 21.9 | 18.7 | 1.17 |
| **7** | e | 26.8 | 54  (54 – 110) | 18 to 333 | 15.9 | 17.8 | 18.3 | 16.2 | 1.13 |
| **8** | Mastiff x | 37.6 | 384  (303 – 420) | 100 to 635 | 21.3 | 24.9 | 23.6 | 18.9 | 1.25 |
| **9** | Mastiff x | 26.3 | 34  (20 – 39) | 17 to 408 | 15.8 | 21.0 | 23.0 | 17.9 | 1.28 |
| **10** | Mastiff x | 34.8 | 44  (21 – 63) | 17 to 312 | 17.0 | 22.3 | 21.6 | 18.8 | 1.15 |

**Table 2. Cuff pressures and volumes to prevent audible air leak using the MOV technique at 15 cm H2O PIP**

| Tube design | ≤30 cm H2O | ≤40 cm H2O | ≤48 cm H2O | >48 cm H2O | Average volume (ml) | Number with audible leaks at cuff pressure >99 cm H2O (out of 10) |
| --- | --- | --- | --- | --- | --- | --- |
| PCK | 3 | 4 | 4 | 6 | 10.15 (3.695) | 2 |
| Melker | 4* | 4 | 4 | 6 | 6.55 (2.535) | 1 |
| H&H | 0 | 0 | 0 | 10* | 7.90 (2.891) | 6 |
| 8.0 | 3 | 7* | 9* | 1 | 6.30 (1.628 | 1 |
| 10.0 | 2 | 6 | 7 | 3 | 6.99 (2.748) | 0 |
| Total | 12 | 21 | 24 | 26 |  |  |

Note, pressures were unknown once exceeding 99 cm H2O.

*Largest number in each category
